# Potential Role of Exosomes in Ischemic Stroke Treatment

**DOI:** 10.3390/biom12010115

**Published:** 2022-01-11

**Authors:** Lingling Jiang, Weiqi Chen, Jinyi Ye, Yilong Wang

**Affiliations:** 1 Department of Neurology, Beijing Tiantan Hospital, Capital Medical University, Beijing 100070, China; linglingjiang712@aliyun.com (L.J.); weiqichen@aliyun.com (W.C.); jinyi_ye@ccmu.edu.cn (J.Y.); 2 Chinese Institute for Brain Research, Beijing 102206, China; 3 China National Clinical Research Center for Neurological Diseases, Beijing 100070, China; 4 Advanced Innovation Center for Human Brain Protection, Capital Medical University, Beijing 100070, China; 5 Beijing Key Laboratory of Translational Medicine for Cerebrovascular Disease, Beijing 100070, China

**Keywords:** exosome, ischemic stroke, microRNA, neurorestoration

## Abstract

Ischemic stroke is a life-threatening cerebral vascular disease and accounts for high disability and mortality worldwide. Currently, no efficient therapeutic strategies are available for promoting neurological recovery in clinical practice, except rehabilitation. The majority of neuroprotective drugs showed positive impact in pre-clinical studies but failed in clinical trials. Therefore, there is an urgent demand for new promising therapeutic approaches for ischemic stroke treatment. Emerging evidence suggests that exosomes mediate communication between cells in both physiological and pathological conditions. Exosomes have received extensive attention for therapy following a stroke, because of their unique characteristics, such as the ability to cross the blood brain–barrier, low immunogenicity, and low toxicity. An increasing number of studies have demonstrated positively neurorestorative effects of exosome-based therapy, which are largely mediated by the microRNA cargo. Herein, we review the current knowledge of exosomes, the relationships between exosomes and stroke, and the therapeutic effects of exosome-based treatments in neurovascular remodeling processes after stroke. Exosomes provide a viable and prospective treatment strategy for ischemic stroke patients.

## 1. Introduction

Ischemic stroke is the leading cause of long-term disability and death in adults worldwide [1,2]. Rehabilitation following ischemic stroke is related to a complex set of courses [3]. Most survivors suffer from a series of neurological dysfunctions and experience modest functional recovery after ischemic stroke. Patients are often unable to be independent in their daily lives, which seriously affects their families socially and economically. Presently, numerous efforts have been devoted to investigating the pathogenesis of this condition and to discovering potential drugs for ischemic stroke by preclinical and clinical studies [4,5,6]. The only useful interventions are thrombolytic therapy with recombinant tissue plasminogen activator (r-tPA) and endovascular thrombectomy. Nevertheless, both approaches have an extremely narrow treatment window (generally within 4.5 h) after the onset of stroke, and few patients benefit from these treatments [7,8,9]. In addition, though various drugs showed a positive impact in pre-clinical studies, none of them has appeared to be able to restore the neurological function [10,11,12,13]. Reliable and effective therapeutic approaches are urgently needed for ischemic stroke patients.

Previous preclinical and early-phase clinical data have confirmed that cell therapy is a safe and promising option for the recovery of neurological function following ischemic stroke [14,15,16,17]. An increasing number of studies suggest that the therapeutic effect of stem cells is mainly mediated by exosomes released from the administered cells [18,19]. Exosomes act as messengers to mediate intercellular communication by delivering biological material, including microRNA and proteins, which plays indispensable roles in physiological and pathological processes [20,21]. Compared with their parent cells, exosomes have a nanoscale size and a lower expression of membrane-bound proteins, which leads to minimal immune response and toxicity in non-immunosuppressed models [22,23,24]. Furthermore, exosomes are stable in the circulation and have the ability to cross the blood–brain barrier (BBB) [25]. These unique characteristics have brought more attention to exosomes. Recent evidence indicates that exosomes can be released into the blood from brain cells responding to stroke, and exosome-based therapy shows beneficial neurorestorative effects following stroke, which are largely mediated by microRNAs [26,27]. In this review, we focus on recent advances about exosome involvement in ischemic stroke and discuss the therapeutic impact and potential applications of exosomes for ischemic stroke treatment.

## 2. Characteristics of Exosomes

**Figure 1:**
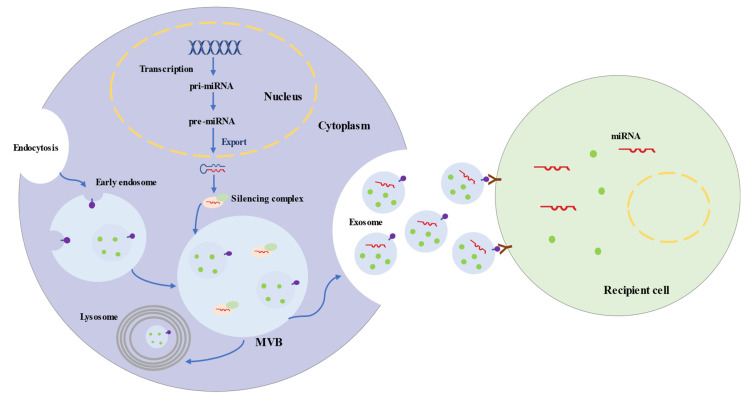
Biogenesis and Secretion Processes of Exosomes and Exosomal microRNAs.

Exosomes represent a subspecies of extracellular vesicles (EVs) with structural size ranging from 30 to 150 nm, released from most cells in all living systems. They exist in various body fluids, such as cerebral spinal fluid, blood, saliva, and urine [28,29,30,31]. Exosomes are initiated by the invagination of the endosomal membrane (Figure 1). Intraluminal vesicles (ILVs) are generated by the inward budding of the endosomal membrane, and early endosomes mature into multivesicular bodies (MVBs). MVBs bind to lysosomes, which results in the degradation of their contents. Additionally, MVBs may fuse with the plasma membrane, leading to the secretion of exosomes [32,33]. The biogenesis of exosomes is strictly regulated by many cell proteins, including Alix, Rab27a, soluble N-ethylmaleimide-sensitive factor attachment protein receptors (SNAREs), and cortactin [34,35,36].

Exosomes are uniformed spheroids with a bilayered lipid membrane. The membrane is rich in cholesterol, sphingomyelin, ceramide, and surface markers from the parent cell, including tetraspanin proteins (CD63, CD81, and CD9), flotillin 1, Alix, and tumor susceptibility gene 101 protein [37,38]. Exosomes carry biologically active substances (proteins, lipids, DNAs, RNAs, and microRNAs), which can mediate cellular communication and modulate a series of physiological processes [39]. Exosome proteins are implicated in antigen presentation, immune reaction, cell binding, and catalytic activity. In addition, proteins (such as brain-derived neurotrophic factor, Zeb2/Axin2) of exosomes participate in brain repair, involving neurogenesis, antiapoptotic processes, and synaptic transmission [40,41]. Of the exosome cargo, microRNAs appear to participate in multiple biological processes [42]. For instance, miRNA-143 is involved in angiogenesis [43], while miR-17-92 cluster and miR-26a are related to neurogenesis and axonal growth signals [44,45].

MicroRNAs are short single-stranded noncoding RNAs and generally consist of 20–25 nucleotides. MicroRNAs are transcribed by RNA polymerase II as primary microRNAs (pri-miRNAs) (Figure 1). The mature microRNA sequence is embedded in the hairpin of pri-miRNA. Following transcription, pri-miRNAs are cleaved into ~65 nt precursor microRNAs (pre-miRNAs) by microprocessor. Pre-miRNAs are transferred into the cytoplasm and processed into double-stranded RNAs, which are 20–25 nt long [46,47]. Ultimately, one strand integrates into a silencing complex and is loaded into MVBs. Along with exosomes, mature microRNAs are released into recipient cells, which are involved in regulating post-transcriptional gene expression and modulating a variety of cellular and molecular pathways [48,49].

Emerging studies have demonstrated that exosomes are involved in the modulation of physiological and pathological processes after ischemic stroke and contribute to brain remodeling by transferring of their cargo. Hence, exosomes have been considered as promising biomarkers for the early diagnosis and prognosis of ischemic stroke and as perspective drugs for the treatment of ischemic stroke [50,51].

## 3. Roles of Exosomes in Ischemic Stroke

### 3.1. Exosomes and Ischemic Stroke Diagnosis

In the central nervous system, exosomes derived from brain cells play significant roles in regulating normal physiological process and responding to acute brain injury [52]. Brain cells, including neurons, microglia, oligodendrocytes, astrocytes, endothelial cells, and pericytes, communicate with each other via their exosomes and exosomal cargos to regulate brain functions, from antioxidation to BBB integrity maintenance and synaptic function [53,54]. Following injury, exosomes are generated by brain cells and evoke diverse responses. Some exosomes seem to have beneficial effects in neuroprotection and neurological recovery. However, some exosomes also have adverse impacts involving neurodegeneration and neuroinflammation [55,56]. Moreover, these exosomes can pass through the BBB and circulate in the peripheral blood and cerebrospinal fluid and could be excellent noninvasive biomarkers for ischemic stroke diagnosis and prognosis [57,58]. Recent studies have detected many components in circulating exosomes which could serve as biomarkers for ischemic stroke, particularly, microRNAs (Table 1) [59,60].

A clinical study indicated that the expression of serum exosomal miR-9 and miR-124 was increased in patients with ischemic stroke and was also positively associated with infarct volume, serum IL-6 concentration, and National Institutes of Health Stroke Scale (NIHSS) scores [59]. These two exosomal microRNAs are considerable biomarkers for diagnosing ischemic stroke and evaluating the degree of ischemic injury [59,61]. Other studies found that circulating exosomal miR-223 and miR-134 were obviously upregulated in acute ischemic stroke patients, strongly associated with NIHSS scores and the expression of IL-6 and high-sensitivity C-reactive protein, and correlated with the occurrence, severity, and worse prognosis of acute ischemic stroke [60,62].

Furthermore, plasma exosomal miR-422a and miR-125b-2-3p were differentially expressed in patients in the acute phase and subacute phase of stroke. They may act as blood-based biomarkers for the diagnosis and monitoring of ischemic stroke. Their combination may be suitable for identifying the ischemic stroke stage [63]. Plasma exosomal miR21-5p and miR-30a-5p in combination were also suggested to be ideal biomarkers for the diagnosis of ischemic stroke and for distinguishing the phase of ischemic stroke [64]. In addition, stroke patients expressed increased levels of miR-17-5p, miR-20b-5p, miR-93-5p, and miR-27b-3p, while patients with cerebral small vessel disease showed the highest miRNAs levels [65]. Patients with cortical–subcortical ischemic stroke showed a lower level of miR-15a, miR-100, miR-339, and miR-424 compared with patients with subcortical ischemic stroke [66]. Preclinical studies indicated that miR-122-5p and miR-300-3p may be used as biomarkers of transient ischemic attack [67]. In summary, exosomes have a critical diagnosis value for stroke and need to be further researched for their potential as biomarkers. The identification of pivotal microRNAs involved in neurorestoration after stroke may contribute to discovering therapeutic targets.

**Table 1:** Exosomal microRNAs as Biomarkers in the Diagnosis of Ischemic Stroke.

microRNAs	Expression in IS	Sources	Models	Outcomes	References
miR-9	upregulation	serum	Human	NIHSS score, infarct volume, serum IL-6	[59]
miR-124	upregulation	serum	Human	NIHSS score, infarct volume, serum IL-6	[59]
miR-223	upregulation	serum	Human	NIHSS score, infarct volume, 3-month mRS, stroke occurrence	[60]
miR-134	upregulation	serum	Human	NIHSS score, infarct volume, serum IL-6, hs-CRP	[62]
miR-422a	upregulation in acute phase downregulation in subacute phase	plasma	Human	different stages of IS	[63]
miR-125-2-3p	downregulation	plasma	Human	different stages of IS	[63]
miR-21-5p	upregulation in subacute phase upregulation in recovery phase	plasma	Human	different stages of IS	[64]
miR-30a-5p	upregulation in hyperacute phase downregulation in acute phase	plasma	Human	different stages of IS	[64]
miR-17-5p	upregulation	serum	Human	subtypes of stroke	[65]
miR-20b-5p	upregulation	serum	Human	subtypes of stroke	[65]
miR-27b-3p	upregulation	serum	Human	subtypes of stroke	[65]
miR-93-5p	upregulation	serum	Human	subtypes of stroke	[65]
miR-15a	downregulation	serum	Human	subgroups of stroke	[66]
miR-100	downregulation	serum	Human	subgroups of stroke	[66]
miR-339	downregulation	serum	Human	subgroups of stroke	[66]
miR-424	downregulation	serum	Human	subgroups of stroke	[66]
miR-122-5p	downregulation	plasma	Rat	different stages of IS	[67]
miR-300-3p	upregulation	plasma	Rat	different stages of IS	[67]
miR-126	downregulation	serum	Rat	different stages of IS	[61]

IS, ischemic stroke; NIHSS, National Institutes of Health Stroke Scale; hs-CRP, high-sensitivity C-reactive protein.

### 3.2. Exosomes and Ischemic Stroke Treatment

Multiple studies demonstrated that cell-based therapy is an excellent method to promote functional outcomes after ischemic stroke, especially if based on mesenchymal stem cells (MSCs) [68,69,70,71]. Exosomes play a significant role in the paracrine effects of stem cells [72,73]. Exosomes from stem cells show low immunogenicity, low tumorigenicity, high transportation efficiency, innate stability, and the capacity to cross the BBB [74,75,76]. They have demonstrated beneficial effects by improving functional recovery after ischemic stroke, because of their ability to enhance brain plasticity [77,78].

Clinical evaluation of exosome therapeutics remains extremely limited, but promising efficacy has been observed in animal ischemic stroke models [79]. Doeppner et al. showed that exosomes from bone marrow MSCs (BMMSCs) efficiently reduced peripheral immunosuppression, enhanced neurovascular regeneration, and improved the motor function 4 weeks after ischemia [80]. MCAO (middle cerebral artery occlusion) rats achieved better results after with intravenous infusion of exosomes in foot fault and modified neurologic severity scores, compared to the PBS group [77]. Exosome treatment post stroke promoted neurite remodeling, angiogenesis, and neurogenesis [77]. Therapy based on exosomes from adipose-derived MSCs (ADMSCs) could reduce the brain infarct zone, improve the recovery of neurological function, and enhance fiber tract integrity and white matter repair in rats after stroke [81,82]. In addition to MSCs-derived exosomes, exosomes released from other cell types, including astrocytes and brain endothelial cells, also contribute to neuroprotective effects after stroke [83,84]. Zhou et al. indicated that astrocyte-derived exosomes could inhibit the expression of TNF-α, IL-6, and IL-1β and ameliorate neuronal damage by suppressing autophagy [83]. Exosomes extracted from endothelial cells contain high levels of miR-126 [84]. Treatment with these exosomes improved the neurological and cognitive functions. Exosomes released from neural stem cells reduced infarct volume and improved function outcome following stroke [85].

Numerous research studies illustrated that exosomes modulate the recipient cells and the rehabilitation process after stroke primarily via microRNAs (Table 2). Xin et al. revealed that exosomes mediated miR-133b transfer to astrocytes and neurons, subsequently enhancing neurite outgrowth and promoting functional recovery after ischemic injury [86,87,88]. Similarly, exosomes enriched with miR-17-92 cluster showed robust effects on neurological function rehabilitation and neural plasticity by modulating the PTEN/Akt/mTOR signaling pathway [89,90]. Another study found that miR-138-5p-enriched exosomes alleviated neurological impairment by accelerating the proliferation of astrocytes and suppressing inflammation by targeting lipocalin 2 in ischemic stroke mice [91]. Furthermore, miR-30d-5p- and miR-223-3p-enhanced exosomes could attenuate cerebral ischemia injury by inhibiting M1 polarization of microglia [92,93]. Exosomes with miR-1906 overexpression downregulated the TLR4 level and enhanced neuroprotection in ischemic mice [94]. miR-132-3p promoted the beneficial effects of exosomes, reducing cerebral vascular ROS production, BBB dysfunction, and brain injury [95]. In addition, miR-21-3p, miR-134, miRNA-184, and miRNA-210 in exosomes were also essential for the prevention of ischemic injury [96,97,98]. Therefore, tailored exosomes with an optimal beneficial microRNA content may maximize their therapeutic potential for ischemic stroke or other neurological disorders. These emerging data highlight the importance of exosomes and their cargos, in particular miRNAs, for brain-remodeling processes.

## 4. Potential Therapeutic Effects of Exosomes in Ischemic Stroke

**Figure 2:**
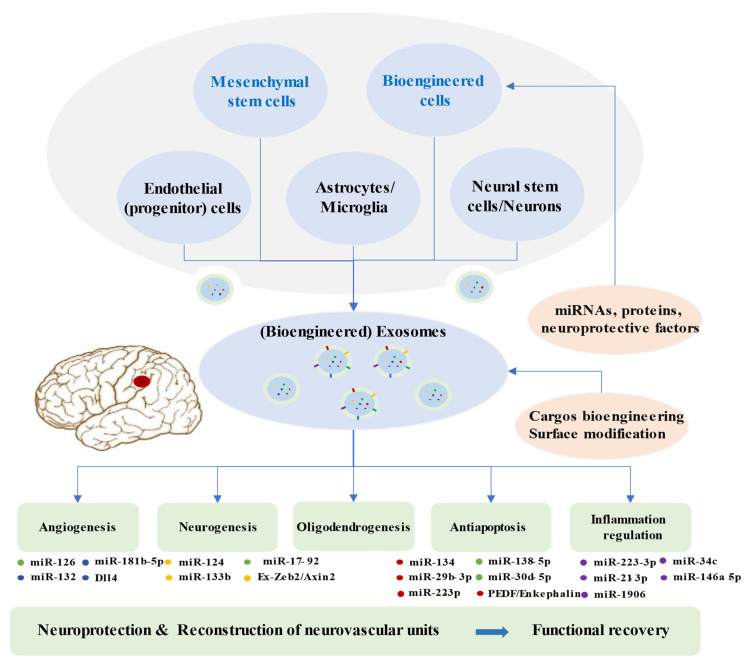
Brain Restoration Processes Regulated by Exosomes after Ischemic Stroke.

Brain restoration after ischemic stroke involves a series of highly interactive processes, including angiogenesis, neurogenesis, oligodendrogenesis, anti-apoptosis, and immune responses, which together accelerate the reconstruction of neurovascular units and neurological recovery [109,110,111]. Recent experiments illustrated that the synthesis and secretion of exosomes and their contents are significantly altered after ischemic events, which may suggest potential targets of the disease [52]. An increasing number of preclinical studies indicated that exosomes from stem cells mediate beneficial effects in ischemic repair by amplifying endogenous brain repair processes (Figure 2) [73,112].

Exosomes derived from multiple cells, including mesenchymal stem cells, endothelial progenitor cells, endothelial cells, astrocytes, microglia, neural stem cells, neuron, and bioengineered cells, can improve the reconstruction of neurovascular units and functional recovery through angiogenesis, neurogenesis, oligodendrogenesis, anti-apoptotic mechanisms, and inflammation regulation following stroke. Exosomes exert beneficial effects in the repair processes mainly via miRNAs. Bioengineered exosomes with cargo or surface modifications will enhance the therapeutic effects.

### 4.1. Angiogenesis

In response to insufficient blood supply after ischemic stroke, the administration of agents to pharmacologically facilitate angiogenesis and restore the blood flow is a common approach. The administration of exosomes with abundant content involving miRNAs, proteins, and lipids may be a novel choice. BMMSCs-derived exosomes were observed to improve the proliferation of cerebral endothelial cells in ischemic animal models [77,98]. miR-126 targeted vascular cell adhesion protein 1 (VCAM-1) and regulated endothelial cell function and angiogenesis [113]. Exosomal miR-126 was downregulated after oxygen–glucose deprivation in brain endothelial cells and in mice after MCAO [61]. Bihl et al. revealed that exosomes obtained from endothelial progenitor cells (EPCs) promoted angiogenesis and neurogenesis in diabetic ischemic stroke mice. Enrichment of miR-126 showed an enhancement of the therapeutic efficacy of EPC-derived exosomes [100]. Moreover, exosomes secreted by adipose-derived stem cells were rich in miRNA-181b-5p. They showed beneficial effects in regulating angiogenesis via suppressing transient receptor potential melastatin 7 (TRPM7) after stroke [102]. Neuron-derived exosomes could deliver miR-132 to endothelial cells to govern cerebral vascular integrity [54]. In addition, the Dll4–Notch signaling pathway in endothelial cells and pericytes is crucial for angiogenesis and BBB integrity. Exosomes derived from human microvascular endothelial cells containing Dll4 proteins regulated angiogenesis. Further research in stroke models is required [114].

### 4.2. Neurogenesis

Neurogenesis together with angiogenesis are vital processes in the recovery from ischemic stroke. Various studies have shown that exosome-based therapy contributes to alterations of neural stem cells and promotes neurogenesis. Exosomes extracted from BMMSCs could promote the proliferation of cerebral neurons after ischemic injury [77]. miR-124 is abundantly expressed in brain tissue and performs a crucial role in neurogenesis; its overexpression can result in neuronal differentiation [115]. The expression of miR-124 was upregulated in ischemic areas following MCAO [116]. Yang et al. revealed that miR-124-loaded exosomes alleviate ischemic injury by facilitating neural progenitor cells differentiation into neuronal lineages [103,104]. Furthermore, MSCs-harvested exosomes with elevated miR-17-92 cluster targeted to the PTEN/Akt pathway in recipient cells led to reduced PTEN and increased phosphorylation of Akt and mTOR, eventually increasing newly generated neurons, neuroplasticity, and oligodendrogenesis in MCAO rats [90]. Exosomes originating from MSCs mediated miR-133b delivery to neurons and astrocytes, which led to the downregulation of CTGF and the secondary release of exosomes from astrocytes, subsequently contributing to neurite outgrowth [86,87,88]. Additionally, Ex-Zeb2/Axin2-enriched exosomes from BMMSCs decreased the expression of SOX10, endothelin-3/EDNRB, and Wnt/β-catenin in MCAO rats, finally improving neuroplasticity and functional recovery [41]. In summary, the utilization of exosomes with active components could be part of a scheme to enhance neurogenesis and activate neuroplasticity, improving neurological recovery after ischemic stroke.

### 4.3. Anti-Apoptosis

Apoptosis is widely involved in the pathogenesis of ischemic stroke and has become an essential target for intervention [117]. Increasing studies have indicated that cell-derived exosomes could inhibit apoptosis and contribute to alleviating cerebral injury in ischemic models [83,92]. Exosomes from different cells demonstrated neuroprotective potential in ischemia-induced neuronal death [105,118,119,120]. MiR-134 was involved in modulating neuronal cell death following ischemia–reperfusion injury [121]. BMMSCs-generated exosomes suppressed oligodendrocyte apoptosis through downregulating the caspase-8 apoptosis pathway via miR-134, which could be a novel therapeutic target for ischemic injury treatment [97]. Neutrophil gelatinase-associated lipocalin (LCN2) is highly expressed after ischemic stroke and is involved in neuron death and brain injury. miR-138-5p is the negatively regulatory factor for LCN2. BMMSCs-released exosomes with overexpressed miR-138-5p downregulated LCN2, caspase-3, and Bax levels, inhibited apoptosis of astrocytes injured by OGD, and reduced neuron injury following ischemia [91]. miR-30d-5p was downregulated in ischemic models. miR-30d-5p-enhanced exosomes showed protective effect against neuronal apoptosis [92]. miR-22-3p in exosomes had beneficial effects in reducing apoptosis and cerebral ischemic injury through the KDM6B/BMP2/BMF axis [106]. Exosomes extracted from endothelial cells protected nerve cells from ischemia–reperfusion injury, partly via inhibiting the apoptosis pathway related to caspase-3, Bax, and Bcl-2 [122]. Furthermore, ADMSCs-derived exosomes with pigment epithelium-derived factor (PEDF) overexpression prevented cerebral ischemia–reperfusion injury in stroke rats through regulating apoptotic factors and activating autophagy [123]. Enkephalin delivery via exosomes released from BMMSCs decreased the expression of p53, caspase-3, and NO, increased neuronal density, and accelerated neurological recovery after rat stroke [124]. In a word, exosomes play a crucial role in anti-apoptosis following ischemic stroke, which is worthy of further exploration.

### 4.4. Inflammation

Inflammation is one of the crucial pathogenic mechanisms of cerebral ischemia, leading to secondary injury to the brain. MSCs-derived exosomes could ameliorate acute ischemic or ischemia–reperfusion injury by regulating anti-inflammatory molecules (IL-4 and IL-10) and pro-inflammatory cytokines (IL-6, TNF-α, and IL-1β), inhibiting microglial inflammation [92]. miR-30d-5p-enhanced exosomes showed a significant effect in modulating microglial phenotypes and in reducing the levels of inflammatory cytokines [92]. Zhao et al. illustrated that exosomes released from BMMSCs exerted anti-inflammatory effects through modulating CysLT2R-ERK1/2-mediated microglia M1 polarization via miR-223-3p [93,99]. Liu et al. reported that BMMSCs-derived exosomes attenuated ischemia–reperfusion injury via the inhibition of NLRP3 inflammasome-mediated inflammation and pyroptosis [125]. ADMSCs-derived exosomes suppressed inflammation and apoptosis via miR-21-3p reduction and MAT2B upregulation in hypoxia/reoxygenation-treated cells [96]. Moreover, exosomes enriched with miR-138-5p or miR-1906 suppressed pro-inflammatory signaling cascades and inhibited inflammatory responses, thereby enhancing rehabilitation following stroke [91,94]. In addition, miR-126 level was reduced in both ischemic patients and animal models of ischemia. miR-126-rich exosomes suppressed neuroinflammation, promoted neurogenesis, and improved functional recovery after stroke [101]. microRNA-34c in astrocyte-released exosomes exerted a neuroprotective effect by downregulating TLR7 and NF-κB/MAPK pathways [107]. miR-146a-5p derived from human umbilical MSC exosomes inhibited microglial-mediated neuroinflammation via the IRAK1/TRAF6 pathway [108]. Accordingly, anti-inflammation is a pivotal mechanism against ischemic injury; targeting specific exosomes related to it may be beneficial.

## 5. Advantages and Modifications of Exosomes for the Therapy of Ischemic Stroke

Exosomes play crucial roles in paracrine pathways of cell therapy and have several advantages over whole cells. Exosomes have minimal oncogenicity, immunogenicity, and toxicity. Abundant exosomes can be produced from a small number of cells and can be stored stably. Moreover, exosomes can cross the BBB and function better than whole cells in the brain [126]. As a consequence, treatments based on exosomes have become a new approach for the treatment of neurological diseases.

Based on the characteristics of exosomes, appropriate modifications of exosomes lead to better clinical efficacy and provide more treatment options. Exosomes mediate therapeutic effects by transferring their cargos, especially microRNAs, thus modulating various pathways [27]. Consequently, engineering exosomes by enriching them with modifying microRNAs can more efficiently activate remodeling and protective pathways within the central nervous system. Compared with normal MSCs-derived exosomes, miR-133b-overexpressing, miR-17-92 cluster-enriched, or miR138-5p-filled MSCs-exosomes improved brain remodeling and functional recovery after stroke [88,90,91]. The directional manipulation of two or more main miRNAs in exosomes from stem cells can potentially enhance the curative effects of exosome. Overexpression of PEDF in exosomes had a good impact on the suppression of apoptosis and ischemic injury [123]. Exosomes highly expressing hepatocyte growth factor (HGF), brain-derived neurotrophic factor (BDNF), and vascular endothelial growth factor (VEGF) may also have therapeutic potential. Furthermore, 98% of all small-molecule drugs cannot pass through the BBB and be efficiently delivered into the brain [127]. Exosomes will be appropriate for delivering pharmacological agents (such as curcumin and enkephalin) and result in considerable therapeutic impacts [124,128].

Additionally, modifications of exosomes surface could further help to enhance specific cell targeting. Rabies virus glycoprotein (RVG) was engineered to bind exosomes when combined with protein lysosome-associated membrane glycoprotein 2b (Lamp2b), which allowed neuron-specific targeting [129]. Yang et al. found that RVG-exosomes could efficiently transfer miR-124 to the infarct location and enhance neuronal protection after ischemic damage [103]. Engineered exosomes conjugated with c (RGDyK) [cyclo (Arg–Gly–Asp–D-Tyr–Lys)] also could target the lesion site of ischemic brain. cRGD–Exo loaded with curcumin led to the inhibition of inflammation and cellular apoptosis [128]. The fusion protein RGD–C1C2 (Arg–Gly–Asp acid 4C peptide fused to lactadherin) bound to exosomes targeted a lesion in ischemic brain and suppressed inflammation [130]. Nevertheless, the modification of exosomes for stroke treatment needs in depth research in the future.

## 6. Conclusions and Future Perspectives

Ischemic stroke is a leading cause of morbidity and mortality worldwide. Early diagnosis and treatment are the main challenges in clinical practice. Exosomes are released from almost all living cells and play significant roles in intercellular communication. Exosomes are deemed powerful biomarkers for the diagnosis of stroke, and exosomes therapies are efficient approaches to improve brain repair through delivering pharmacological agents or genes after stroke. However, exosomes research remains in its initial stage, particularly for ischemic stroke; no adequate information is available to translate exosomes treatment into clinical practice. A better understanding of exosomes will be beneficial to stroke diagnosis and therapy. Exosomes therapy still has many limitations and presents many challenges. Firstly, the content of exosomes including proteins, miRNAs, and lipids varies, depending on the donor cells, the conditions of cell culture, and exosome extraction. The optimization of operational procedures is necessary, and the characterization of exosome cargos mediating therapeutic effects is warranted. Secondly, new costless techniques for obtaining high-purity exosomes in large amounts need to be developed. Lastly, the extension of exosomes’ half-life and the improvement of their targeting ability require attention for their application in medicine. In conclusion, clinical-grade exosomes appear to be novel promising therapeutic approaches and require further studies.

## Figures and Tables

**Table 2:** Exosomal microRNAs have been Used to Treat Ischemic Stroke.

microRNAs	Models	Sources	Proposed Effects	Involved Pathway	References
miR-133b	MCAO-rat	MSC	Neural remodeling	CTGF	[86,87,88]
miR-17-92 cluster	MCAO-rat	MSC	Neural remodeling	PTEN/Akt/mTOR pathway	[90]
miR-138-5p	MCAO-mouse OGD-astrocyte	MSC	Anti-inflammation Anti-apoptosis	Lipocalin 2	[91]
miR-30d-5p	MCAO-rat OGD-microglia	MSC	Anti-inflammation Anti-apoptosis	Beclin-1/Atg5	[92]
miR-223-3p	MCAO-rat OGD-microglia	MSC	Anti-inflammation	CysLT2R-ERK1/2	[93,99]
miR-1906	MCAO-mouse OGD-neuron	MSC	Anti-inflammation	TLR4	[94]
miR-132-3p	MCAO-mouse endothelial cell	MSC	BBB protection Reduce vascular ROS	PI3K/Akt/eNOS pathway	[95]
miR-21-3p	MCAO-rat	MSC	BBB protection Anti-inflammation Anti-apoptosis	MAT2B	[96]
miR-134	OGD-oligodendrocyte	MSC	Anti-apoptosis	Caspase-8	[97]
miR-184	MCAO-rat	MSC	Neurogenesis Angiogenesis	----	[98]
miR-210	MCAO-rat	MSC	Neurogenesis Angiogenesis	ephrin-A3	[98]
miR-126	MCAO-mouse	EPC	Neurogenesis Angiogenesis Anti-apoptosis	Caspase-3VEGFR2	[100,101]
miR-181b-5p	OGD-endothelial cell	MSC	Angiogenesis	TTRPM7	[102]
miR-132	zebrafish larvaeendothelial cell	Neuron	Angiogenesis	Cdh5/eEF2K	[54]
miR-124	Photothrombosis mouse	MSC	Neurogenesis	GLI3 STAT3	[103]
	MCAO-mouse OGD-neuron	M2 microglia	Anti-apoptosis	USP14	[104]
miR-137	MCAO-mouse OGD-neuron	Microglia	Anti-apoptosis	Notch1	[105]
miR-22-3p	MCAO-rat OGD-neuron	MSC	Anti-apoptosis	KDM6B/BMP2/BMF axis	[106]
miR-34c	MCAO-rat OGD-neuroblastoma cells	Astrocyte	Anti-inflammation Anti-apoptosis	TLR7 and NFκB/MAPK pathways	[107]
miR-146a-5p	MCAO-mouse OGD-microglia	MSC	Anti-inflammation	IRAK1/TRAF6 pathway	[108]

MCAO, middle cerebral artery occlusion; MSC, mesenchymal stromal cells; CTGF, connective tissue growth factor; PTEN, phosphatase and tensin homolog; Akt, protein kinase B; mTOR, mechanistic target of rapamycin; OGD, oxygen and glucose deprivation; CysLT2R, cysteinyl leukotriene receptor 2; ERK, extracellular regulated protein kinases; BBB, blood–brain barrier; TLR4, Toll-like receptor 4; ROS, reactive oxygen species; PI3K, phosphatidylinositol 3 kinase; eNOS, endothelial nitric oxide synthesis; MAT2B, methionine adenosyltransferase 2B; EPC, endothelial progenitor cells; VEGFR2, vascular endothelial growth factor receptor 2; TRPM7, transient receptor potential melastatin 7; Cdh5, cadherin 5; eEF2K, eukaryotic elongation factor 2 kinase; GLI3, gli family zinc finger 3; STAT3, signal transducer and activator of transcription 3; USP14, ubiquitin-specific protease 14; KDM6B, lysine demethylase 6B; BMP2, bone morphogenetic protein 2; BMF, bcl2 modifying factor; IRAK1, interleukin 1 receptor associated kinase 1; TRAF6, TNF receptor associated factor 6.

## Data Availability

Not applicable.

## References

[1] Feigin V.L., Krishnamurthi R.V., Parmar P., Norrving B., Mensah G.A., Bennett D.A., Barker-Collo S., Moran A.E., Sacco R.L., Truelsen T. (2015). Update on the Global Burden of Ischemic and Hemorrhagic Stroke in 1990–2013: The GBD 2013 Study. Neuroepidemiology.

[2] Wang W., Jiang B., Sun H., Ru X., Sun D., Wang L., Wang L., Jiang Y., Li Y., Wang Y. (2017). Prevalence, Incidence, and Mortality of Stroke in China: Results from a Nationwide Population-Based Survey of 480 687 Adults. Circulation.

[3] Zhang Z.G., Chopp M. (2015). Promoting brain remodeling to aid in stroke recovery. Trends. Mol. Med..

[4] Langhorne P., Bernhardt J., Kwakkel G. (2011). Stroke rehabilitation. Lancet.

[5] Moretti A., Ferrari F., Villa R.F. (2015). Neuroprotection for ischaemic stroke: Current status and challenges. Pharmacol. Ther..

[6] Campbell B.C.V., De Silva D.A., Macleod M.R., Coutts S.B., Schwamm L.H., Davis S.M., Donnan G.A. (2019). Ischaemic stroke. Nat. Rev. Dis. Primers..

[7] Jovin T.G., Chamorro A., Cobo E., de Miquel M.A., Molina C.A., Rovira A., San Roman L., Serena J., Abilleira S., Ribo M. (2015). Thrombectomy within 8 hours after symptom onset in ischemic stroke. N. Engl. J. Med..

[8] Zerna C., Thomalla G., Campbell B.C.V., Rha J.H., Hill M.D. (2018). Current practice and future directions in the diagnosis and acute treatment of ischaemic stroke. Lancet.

[9] Powers W.J., Rabinstein A.A., Ackerson T., Adeoye O.M., Bambakidis N.C., Becker K., Biller J., Brown M., Demaerschalk B.M., Hoh B. (2019). Guidelines for the Early Management of Patients With Acute Ischemic Stroke: 2019 Update to the 2018 Guidelines for the Early Management of Acute Ischemic Stroke: A Guideline for Healthcare Professionals From the American Heart Association/American Stroke Association. Stroke.

[10] Shuaib A., Lees K.R., Lyden P., Grotta J., Davalos A., Davis S.M., Diener H.C., Ashwood T., Wasiewski W.W., Emeribe U. (2007). NXY-059 for the treatment of acute ischemic stroke. N. Engl. J. Med..

[11] Davalos A., Alvarez-Sabin J., Castillo J., Diez-Tejedor E., Ferro J., Martinez-Vila E., Serena J., Segura T., Cruz V.T., Masjuan J. (2012). Citicoline in the treatment of acute ischaemic stroke: An international, randomised, multicentre, placebo-controlled study (ICTUS trial). Lancet.

[12] Albers G.W., von Kummer R., Truelsen T., Jensen J.K., Ravn G.M., Gronning B.A., Chabriat H., Chang K.C., Davalos A.E., Ford G.A. (2015). Safety and efficacy of desmoteplase given 3-9 h after ischaemic stroke in patients with occlusion or high-grade stenosis in major cerebral arteries (DIAS-3): A double-blind, randomised, placebo-controlled phase 3 trial. Lancet Neurol..

[13] von Kummer R., Mori E., Truelsen T., Jensen J.S., Gronning B.A., Fiebach J.B., Lovblad K.O., Pedraza S., Romero J.M., Chabriat H. (2016). Desmoteplase 3 to 9 Hours After Major Artery Occlusion Stroke: The DIAS-4 Trial (Efficacy and Safety Study of Desmoteplase to Treat Acute Ischemic Stroke). Stroke.

[14] Gervois P., Wolfs E., Ratajczak J., Dillen Y., Vangansewinkel T., Hilkens P., Bronckaers A., Lambrichts I., Struys T. (2016). Stem Cell-Based Therapies for Ischemic Stroke: Preclinical Results and the Potential of Imaging-Assisted Evaluation of Donor Cell Fate and Mechanisms of Brain Regeneration. Med. Res. Rev..

[15] Nagpal A., Choy F.C., Howell S., Hillier S., Chan F., Hamilton-Bruce M.A., Koblar S.A. (2017). Safety and effectiveness of stem cell therapies in early-phase clinical trials in stroke: A systematic review and meta-analysis. Stem Cell Res. Ther..

[16] Levy M.L., Crawford J.R., Dib N., Verkh L., Tankovich N., Cramer S.C. (2019). Phase I/II Study of Safety and Preliminary Efficacy of Intravenous Allogeneic Mesenchymal Stem Cells in Chronic Stroke. Stroke.

[17] Ouyang Q., Li F., Xie Y., Han J., Zhang Z., Feng Z., Su D., Zou X., Cai Y., Zou Y. (2019). Meta-Analysis of the Safety and Efficacy of Stem Cell Therapies for Ischemic Stroke in Preclinical and Clinical Studies. Stem Cells Dev..

[18] Wang W., Li Z., Feng J. (2018). The potential role of exosomes in the diagnosis and therapy of ischemic diseases. Cytotherapy.

[19] Zhang Z.G., Buller B., Chopp M. (2019). Exosomes - beyond stem cells for restorative therapy in stroke and neurological injury. Nat. Rev. Neurol..

[20] Wang M.M., Feng Y.S., Tan Z.X., Xing Y., Dong F., Zhang F. (2020). The role of exosomes in stroke. Mol. Biol. Rep..

[21] Valadi H., Ekström K., Bossios A., Sjöstrand M., Lee J.J., Lötvall J.O. (2007). Exosome-mediated transfer of mRNAs and microRNAs is a novel mechanism of genetic exchange between cells. Nat. Cell Biol..

[22] de Abreu R.C., Fernandes H., da Costa Martins P.A., Sahoo S., Emanueli C., Ferreira L. (2020). Native and bioengineered extracellular vesicles for cardiovascular therapeutics. Nat. Rev. Cardiol..

[23] Aminzadeh M.A., Rogers R.G., Fournier M., Tobin R.E., Guan X., Childers M.K., Andres A.M., Taylor D.J., Ibrahim A., Ding X. (2018). Exosome-Mediated Benefits of Cell Therapy in Mouse and Human Models of Duchenne Muscular Dystrophy. Stem Cell Rep..

[24] Lou G., Chen Z., Zheng M., Liu Y. (2017). Mesenchymal stem cell-derived exosomes as a new therapeutic strategy for liver diseases. Exp. Mol. Med..

[25] Khan H., Pan J.J., Li Y., Zhang Z., Yang G.Y. (2021). Native and Bioengineered Exosomes for Ischemic Stroke Therapy. Front. Cell Dev. Biol..

[26] Nasirishargh A., Kumar P., Ramasubramanian L., Clark K., Hao D., Lazar S.V., Wang A. (2021). Exosomal microRNAs from mesenchymal stem stromal cells Biology and applications in neuroprotection. World J. Stem Cells.

[27] Ghoreishy A., Khosravi A., Ghaemmaghami A. (2019). Exosomal microRNA and stroke: A review. J. Cell Biochem..

[28] Tian Y., Fu C., Wu Y., Lu Y., Liu X., Zhang Y. (2021). Central Nervous System Cell-Derived Exosomes in Neurodegenerative Diseases. Oxid. Med. Cell Longev..

[29] Mi B., Chen L., Xiong Y., Yan C., Xue H., Panayi A.C., Liu J., Hu L., Hu Y., Cao F. (2020). Saliva exosomes-derived UBE2O mRNA promotes angiogenesis in cutaneous wounds by targeting SMAD6. J. Nanobiotechnology.

[30] Lässer C., Alikhani V.S., Ekström1 K., Eldh M., Paredes P.T., Bossios A., Sjöstrand M., Gabrielsson S., Lötvall J., Valadi H. (2011). Human saliva, plasma and breast milk exosomes contain RNA uptake by macrophages. J. Transl. Med..

[31] Street J.M., Koritzinsky E.H., Glispie D.M., Star R.A., Yuen P.S. (2017). Urine Exosomes: An Emerging Trove of Biomarkers. Adv. Clin. Chem..

[32] Colombo M., Raposo G., Théry C. (2014). Biogenesis, secretion, and intercellular interactions of exosomes and other extracellular vesicles. Annu. Rev. Cell Dev. Biol..

[33] Kowal J., Tkach M., Théry C. (2014). Biogenesis and secretion of exosomes. Curr. Opin. Cell Biol..

[34] Fader C.M., Sánchez D.G., Mestre M.B., Colombo M.I. (2009). TI-VAMP/VAMP7 and VAMP3/cellubrevin: Two v-SNARE proteins involved in specific steps of the autophagy/multivesicular body pathways. Biochim. Biophys. Acta..

[35] Sinha S., Hoshino D., Hong N.H., Kirkbride K.C., Grega-Larson N.E., Seiki M., Tyska M.J., Weaver A.M. (2016). Cortactin promotes exosome secretion by controlling branched actin dynamics. J. Cell. Biol..

[36] Baietti M.F., Zhang Z., Mortier E., Melchior A., Degeest G., Geeraerts A., Ivarsson Y., Depoortere F., Coomans C., Vermeiren E. (2012). Syndecan-syntenin-ALIX regulates the biogenesis of exosomes. Nat. Cell Biol..

[37] Pegtel D.M., Gould S.J. (2019). Exosomes. Annu. Rev. Biochem..

[38] Hong S.B., Yang H., Manaenko A., Lu J., Mei Q., Hu Q. (2019). Potential of Exosomes for the Treatment of Stroke. Cell Transplant..

[39] Meldolesi J. (2018). Exosomes and Ectosomes in Intercellular Communication. Curr. Biol..

[40] Xu H., Jia Z., Ma K., Zhang J., Dai C., Yao Z., Deng W., Su J., Wang R., Chen X. (2020). Protective effect of BMSCs-derived exosomes mediated by BDNF on TBI via miR-216a-5p. Med. Sci. Monit..

[41] Wei R., Zhang L., Hu W., Shang X., He Y., Zhang W. (2021). Zeb2/Axin2-Enriched BMSC-Derived Exosomes Promote Post-Stroke Functional Recovery by Enhancing Neurogenesis and Neural Plasticity. J. Mol. Neurosci..

[42] Happel C., Ganguly A., Tagle D.A. (2020). Extracellular RNAs as potential biomarkers for cancer. J. Cancer Metastasis Treat..

[43] Geng T., Song Z.Y., Xing J.X., Wang B.X., Dai S.P., Xu Z.S. (2020). Exosome Derived from Coronary Serum of Patients with Myocardial Infarction Promotes Angiogenesis Through the miRNA-143/IGF-IR Pathway. Int. J. Nanomed..

[44] Xin H., Liu Z., Buller B., Li Y., Golembieski W., Gan X., Wang F., Lu M., Ali M.M., Zhang Z.G. (2021). MiR-17-92 enriched exosomes derived from multipotent mesenchymal stromal cells enhance axon-myelin remodeling and motor electrophysiological recovery after stroke. J. Cereb. Blood Flow Metab..

[45] Ling X., Zhang G., Xia Y., Zhu Q., Zhang J., Li Q., Niu X., Hu G., Yang Y., Wang Y. (2020). Exosomes from human urine-derived stem cells enhanced neurogenesis via miR-26a/HDAC6 axis after ischaemic stroke. J. Cell. Mol. Med..

[46] Treiber T., Treiber N., Meister G. (2019). Regulation of microRNA biogenesis and its crosstalk with other cellular pathways. Nat. Rev. Mol. Cell. Biol..

[47] Ha M., Kim V.N. (2014). Regulation of microRNA biogenesis. Nat. Rev. Mol. Cell. Biol..

[48] Yu X., Odenthal M., Fries J.W. (2016). Exosomes as miRNA Carriers: Formation-Function-Future. Int. J. Mol. Sci..

[49] Saliminejad K., Khorram Khorshid H.R., Soleymani Fard S., Ghaffari S.H. (2019). An overview of microRNAs: Biology, functions, therapeutics, and analysis methods. J. Cell. Physiol..

[50] Zhang Z.G., Chopp M. (2016). Exosomes in stroke pathogenesis and therapy. J. Clin. Invest..

[51] Azizi F., Askari S., Javadpour P., Hadjighassem M., Ghasemi R. (2020). Potential role of exosome in post-stroke reorganization and/or neurodegeneration. EXCLI J..

[52] Zagrean A.M., Hermann D.M., Opris I., Zagrean L., Popa-Wagner A. (2018). Multicellular Crosstalk Between Exosomes and the Neurovascular Unit After Cerebral Ischemia. Therapeutic Implications. Front. Neurosci..

[53] Budnik V., Ruiz-Canada C., Wendler F. (2016). Extracellular vesicles round off communication in the nervous system. Nat. Rev. Neurosci..

[54] Xu B., Zhang Y., Du X.F., Li J., Zi H.X., Bu J.W., Yan Y., Han H., Du J.L. (2017). Neurons secrete miR-132-containing exosomes to regulate brain vascular integrity. Cell Res..

[55] Li J.J., Wang B., Kodali M.C., Chen C., Kim E., Patters B.J., Lan L., Kumar S., Wang X., Yue J. (2018). In vivo evidence for the contribution of peripheral circulating inflammatory exosomes to neuroinflammation. J. Neuroinflammation.

[56] Perez-Gonzalez R., Gauthier S.A., Kumar A., Levy E. (2012). The exosome secretory pathway transports amyloid precursor protein carboxyl-terminal fragments from the cell into the brain extracellular space. J. Biol. Chem..

[57] Datta A., Chen C.P., Sze S.K. (2014). Discovery of prognostic biomarker candidates of lacunar infarction by quantitative proteomics of microvesicles enriched plasma. PLoS ONE.

[58] Otero-Ortega L., Laso-García F., Gómez-de Frutos M., Fuentes B., Diekhorst L., Díez-Tejedor E., Gutiérrez-Fernández M. (2019). Role of Exosomes as a Treatment and Potential Biomarker for Stroke. Transl. Stroke Res..

[59] Ji Q., Ji Y., Peng J., Zhou X., Chen X., Zhao H., Xu T., Chen L., Xu Y. (2016). Increased Brain-Specific MiR-9 and MiR-124 in the Serum Exosomes of Acute Ischemic Stroke Patients. PLoS ONE.

[60] Chen Y., Song Y., Huang J., Qu M., Zhang Y., Geng J., Zhang Z., Liu J., Yang G.Y. (2017). Increased Circulating Exosomal miRNA-223 Is Associated with Acute Ischemic Stroke. Front. Neurol..

[61] Chen F., Du Y., Esposito E., Liu Y., Guo S., Wang X., Lo E.H., Xing C., Ji X. (2015). Effects of Focal Cerebral Ischemia on Exosomal Versus Serum miR126. Transl. Stroke Res..

[62] Zhou J., Chen L., Chen B., Huang S., Zeng C., Wu H., Chen C., Long F. (2018). Increased serum exosomal miR-134 expression in the acute ischemic stroke patients. BMC Neurol..

[63] Li D.B., Liu J.L., Wang W., Li R.Y., Yu D.J., Lan X.Y., Li J.P. (2017). Plasma Exosomal miR-422a and miR-125b-2-3p Serve as Biomarkers for Ischemic Stroke. Curr. Neurovasc. Res..

[64] Wang W., Li D.B., Li R.Y., Zhou X., Yu D.J., Lan X.Y., Li J.P., Liu J.L. (2018). Diagnosis of Hyperacute and Acute Ischaemic Stroke: The Potential Utility of Exosomal MicroRNA-21-5p and MicroRNA-30a-5p. Cerebrovasc. Dis..

[65] van Kralingen J.C., McFall A., Ord E.N.J., Coyle T.F., Bissett M., McClure J.D., McCabe C., Macrae I.M., Dawson J., Work L.M. (2019). Altered Extracellular Vesicle MicroRNA Expression in Ischemic Stroke and Small Vessel Disease. Transl. Stroke Res..

[66] Otero-Ortega L., Alonso-López E., Pérez-Mato M., Laso-García F., Gómez-de Frutos M.C., Diekhorst L., García-Bermejo M.L., Conde-Moreno E., Fuentes B., de Leciñana M.A. (2021). Circulating Extracellular Vesicle Proteins and MicroRNA Profiles in Subcortical and Cortical-Subcortical Ischaemic Stroke. Biomedicines.

[67] Li D.B., Liu J.L., Wang W., Luo X.M., Zhou X., Li J.P., Cao X.L., Long X.H., Chen J.G., Qin C. (2018). Plasma Exosomal miRNA-122-5p and miR-300-3p as Potential Markers for Transient Ischaemic Attack in Rats. Front. Aging Neurosci..

[68] Zhang G., Zhu Z., Wang Y. (2019). Neural stem cell transplantation therapy for brain ischemic stroke Review and perspectives. World J. Stem Cells.

[69] Wang F., Tang H., Zhu J., Zhang J.H. (2018). Transplanting Mesenchymal Stem Cells for Treatment of Ischemic Stroke. Cell Transplant..

[70] Cunningham C.J., Wong R., Barrington J., Tamburrano S., Pinteaux E., Allan S.M. (2020). Systemic conditioned medium treatment from interleukin-1 primed mesenchymal stem cells promotes recovery after stroke. Stem Cell Res. Ther..

[71] Doeppner T.R., Kaltwasser B., Teli M.K., Bretschneider E., Bahr M., Hermann D.M. (2014). Effects of acute versus post-acute systemic delivery of neural progenitor cells on neurological recovery and brain remodeling after focal cerebral ischemia in mice. Cell Death Dis..

[72] Qiu G., Zheng G., Ge M., Wang J., Huang R., Shu Q., Xu J. (2018). Mesenchymal stem cell-derived extracellular vesicles affect disease outcomes via transfer of microRNAs. Stem Cell Res. Ther..

[73] Bang O.Y., Kim E.H. (2019). Mesenchymal Stem Cell-Derived Extracellular Vesicle Therapy for Stroke: Challenges and Progress. Front. Neurol..

[74] Chen C.C., Liu L., Ma F., Wong C.W., Guo X.E., Chacko J.V., Farhoodi H.P., Zhang S.X., Zimak J., Segaliny A. (2016). Elucidation of Exosome Migration across the Blood-Brain Barrier Model In Vitro. Cell Mol. Bioeng..

[75] Zhu X., Badawi M., Pomeroy S., Sutaria D.S., Xie Z., Baek A., Jiang J., Elgamal O.A., Mo X., Perle K. (2017). Comprehensive toxicity and immunogenicity studies reveal minimal effects in mice following sustained dosing of extracellular vesicles derived from HEK293T cells. J. Extracell. Vesicles.

[76] Gowen A., Shahjin F., Chand S., Odegaard K.E., Yelamanchili S.V. (2020). Mesenchymal Stem Cell-Derived Extracellular Vesicles: Challenges in Clinical Applications. Front. Cell. Dev. Biol..

[77] Xin H., Li Y., Cui Y., Yang J.J., Zhang Z.G., Chopp M. (2013). Systemic administration of exosomes released from mesenchymal stromal cells promote functional recovery and neurovascular plasticity after stroke in rats. J. Cereb. Blood Flow Metab..

[78] Gao B., Zhou S., Sun C., Cheng D., Zhang Y., Li X., Zhang L., Zhao J., Xu D., Bai Y. (2020). Brain Endothelial Cell-Derived Exosomes Induce Neuroplasticity in Rats with Ischemia/Reperfusion Injury. ACS Chem. Neurosci..

[79] Huang M., Hong Z., Xiao C., Li L., Chen L., Cheng S., Lei T., Zheng H. (2020). Effects of Exosomes on Neurological Function Recovery for Ischemic Stroke in Pre-clinical Studies: A Meta-analysis. Front. Cell Neurosci..

[80] Doeppner T.R., Herz J., Görgens A., Schlechter J., Ludwig A.K., Radtke S., de Miroschedji K., Horn P.A., Giebel B., Hermann D.M. (2015). Extracellular Vesicles Improve Post-Stroke Neuroregeneration and Prevent Postischemic Immunosuppression. Stem Cells Transl. Med..

[81] Chen K.H., Chen C.H., Wallace C.G., Yuen C.M., Kao G.S., Chen Y.L., Shao P.L., Chen Y.L., Chai H.T., Lin K.C. (2016). Intravenous administration of xenogenic adipose-derived mesenchymal stem cells (ADMSC) and ADMSC-derived exosomes markedly reduced brain infarct volume and preserved neurological function in rat after acute ischemic stroke. Oncotarget.

[82] Otero-Ortega L., Laso-Garcia F., Gomez-de Frutos M.D., Rodriguez-Frutos B., Pascual-Guerra J., Fuentes B., Diez-Tejedor E., Gutierrez-Fernandez M. (2017). White Matter Repair After Extracellular Vesicles Administration in an Experimental Animal Model of Subcortical Stroke. Sci. Rep..

[83] Pei X., Li Y., Zhu L., Zhou Z. (2019). Astrocyte-derived exosomes suppress autophagy and ameliorate neuronal damage in experimental ischemic stroke. Exp. Cell Res..

[84] Venkat P., Cui C., Chopp M., Zacharek A., Wang F., Landschoot-Ward J., Shen Y., Chen J. (2019). MiR-126 Mediates Brain Endothelial Cell Exosome Treatment-Induced Neurorestorative Effects After Stroke in Type 2 Diabetes Mellitus Mice. Stroke.

[85] Webb R.L., Kaiser E.E., Scoville S.L., Thompson T.A., Fatima S., Pandya C., Sriram K., Swetenburg R.L., Vaibhav K., Arbab A.S. (2018). Human Neural Stem Cell Extracellular Vesicles Improve Tissue and Functional Recovery in the Murine Thromboembolic Stroke Model. Transl. Stroke Res..

[86] Xin H., Li Y., Buller B., Katakowski M., Zhang Y., Wang X., Shang X., Zhang Z.G., Chopp M. (2012). Exosome-mediated transfer of miR-133b from multipotent mesenchymal stromal cells to neural cells contributes to neurite outgrowth. Stem Cells.

[87] Xin H., Li Y., Liu Z., Wang X., Shang X., Cui Y., Zhang Z.G., Chopp M. (2013). MiR-133b promotes neural plasticity and functional recovery after treatment of stroke with multipotent mesenchymal stromal cells in rats via transfer of exosome-enriched extracellular particles. Stem Cells.

[88] Xin H., Wang F., Li Y., Lu Q.E., Cheung W.L., Zhang Y., Zhang Z.G., Chopp M. (2017). Secondary Release of Exosomes From Astrocytes Contributes to the Increase in Neural Plasticity and Improvement of Functional Recovery After Stroke in Rats Treated With Exosomes Harvested From MicroRNA 133b-Overexpressing Multipotent Mesenchymal Stromal Cells. Cell Transplant..

[89] Zhang Y., Chopp M., Liu X.S., Katakowski M., Wang X., Tian X., Wu D., Zhang Z.G. (2017). Exosomes Derived from Mesenchymal Stromal Cells Promote Axonal Growth of Cortical Neurons. Mol. Neurobiol..

[90] Xin H., Katakowski M., Wang F., Qian J.Y., Liu X.S., Ali M.M., Buller B., Zhang Z.G., Chopp M. (2017). MicroRNA cluster miR-17-92 Cluster in Exosomes Enhance Neuroplasticity and Functional Recovery After Stroke in Rats. Stroke.

[91] Deng Y., Chen D., Gao F., Lv H., Zhang G., Sun X., Liu L., Mo D., Ma N., Song L. (2019). Exosomes derived from microRNA-138-5p-overexpressing bone marrow-derived mesenchymal stem cells confer neuroprotection to astrocytes following ischemic stroke via inhibition of LCN2. J. Biol. Eng..

[92] Jiang M., Wang H., Jin M., Yang X., Ji H., Jiang Y., Zhang H., Wu F., Wu G., Lai X. (2018). Exosomes from MiR-30d-5p-ADSCs Reverse Acute Ischemic Stroke-Induced, Autophagy-Mediated Brain Injury by Promoting M2 Microglial/Macrophage Polarization. Cell. Physiol. Biochem..

[93] Zhao Y., Gan Y., Xu G., Hua K., Liu D. (2020). Exosomes from MSCs overexpressing microRNA-223-3p attenuate cerebral ischemia through inhibiting microglial M1 polarization mediated inflammation. Life Sci..

[94] Haupt M., Zheng X., Kuang Y., Lieschke S., Janssen L., Bosche B., Jin F., Hein K., Kilic E., Venkataramani V. (2021). Lithium modulates miR-1906 levels of mesenchymal stem cell-derived extracellular vesicles contributing to poststroke neuroprotection by toll-like receptor 4 regulation. Stem Cells Transl. Med..

[95] Pan Q., Kuang X., Cai S., Wang X., Du D., Wang J., Wang Y., Chen Y., Bihl J., Chen Y. (2020). miR-132-3p priming enhances the effects of mesenchymal stromal cell-derived exosomes on ameliorating brain ischemic injury. Stem Cell Res. Ther..

[96] Li C., Fei K., Tian F., Gao C., Yang S. (2019). Adipose-derived mesenchymal stem cells attenuate ischemic brain injuries in rats by modulating miR-21-3p/MAT2B signaling transduction. Croat. Med. J..

[97] Xiao Y., Geng F., Wang G., Li X., Zhu J., Zhu W. (2018). Bone marrow-derived mesenchymal stem cells-derived exosomes prevent oligodendrocyte apoptosis through exosomal miR-134 by targeting caspase-8. J. Cell Biochem..

[98] Moon G.J., Sung J.H., Kim D.H., Kim E.H., Cho Y.H., Son J.P., Cha J.M., Bang O.Y. (2019). Application of Mesenchymal Stem Cell-Derived Extracellular Vesicles for Stroke: Biodistribution and MicroRNA Study. Transl. Stroke Res..

[99] Zhao Y., Gan Y., Xu G., Yin G., Liu D. (2020). MSCs-Derived Exosomes Attenuate Acute Brain Injury and Inhibit Microglial Inflammation by Reversing CysLT2R-ERK1/2 Mediated Microglia M1 Polarization. Neurochem. Res..

[100] Wang J., Chen S., Zhang W., Chen Y., Bihl J.C. (2020). Exosomes from miRNA-126-modified endothelial progenitor cells alleviate brain injury and promote functional recovery after stroke. CNS Neurosci. Ther..

[101] Geng W., Tang H., Luo S., Lv Y., Liang D., Kang X., Hong W. (2019). Exosomes from miRNA-126-modified ADSCs promotes functional recovery after stroke in rats by improving neurogenesis and suppressing microglia activation. Am. J. Transl. Res..

[102] Yang Y., Cai Y., Zhang Y., Liu J., Xu Z. (2018). Exosomes Secreted by Adipose-Derived Stem Cells Contribute to Angiogenesis of Brain Microvascular Endothelial Cells Following Oxygen-Glucose Deprivation In Vitro Through MicroRNA-181b/TRPM7 Axis. J. Mol. Neurosci..

[103] Yang J., Zhang X., Chen X., Wang L., Yang G. (2017). Exosome Mediated Delivery of miR-124 Promotes Neurogenesis after Ischemia. Mol. Ther. Nucleic Acids.

[104] Song Y., Li Z., He T., Qu M., Jiang L., Li W., Shi X., Pan J., Zhang L., Wang Y. (2019). M2 microglia-derived exosomes protect the mouse brain from ischemia-reperfusion injury via exosomal miR-124. Theranostics.

[105] Zhang D., Cai G., Liu K., Zhuang Z., Jia K., Pei S., Wang X., Wang H., Xu S., Cui C. (2021). Microglia exosomal miRNA-137 attenuates ischemic brain injury through targeting Notch1. Aging.

[106] Zhang Y., Liu J., Su M., Wang X., Xie C. (2021). Exosomal microRNA-22-3p alleviates cerebral ischemic injury by modulating KDM6B/BMP2/BMF axis. Stem Cell Res. Ther..

[107] Wu W., Liu J., Yang C., Xu Z., Huang J., Lin J. (2020). Astrocyte-derived exosome-transported microRNA-34c is neuroprotective against cerebral ischemia/reperfusion injury via TLR7 and the NF-κB/MAPK pathways. Brain Res. Bull..

[108] Zhang Z., Zou X., Zhang R., Xie Y., Feng Z., Li F., Han J., Sun H., Ouyang Q., Hua S. (2021). Human umbilical cord mesenchymal stem cell-derived exosomal miR-146a-5p reduces microglial-mediated neuroinflammation via suppression of the IRAK1/TRAF6 signaling pathway after ischemic stroke. Aging.

[109] Dabrowska S., Andrzejewska A., Lukomska B., Janowski M. (2019). Neuroinflammation as a target for treatment of stroke using mesenchymal stem cells and extracellular vesicles. J. Neuroinflammation.

[110] Chavez L.M., Huang S.S., MacDonald I., Lin J.G., Lee Y.C., Chen Y.H. (2017). Mechanisms of Acupuncture Therapy in Ischemic Stroke Rehabilitation: A Literature Review of Basic Studies. Int. J. Mol. Sci..

[111] Kang L., Yu H., Yang X., Zhu Y., Bai X., Wang R., Cao Y., Xu H., Luo H., Lu L. (2020). Neutrophil extracellular traps released by neutrophils impair revascularization and vascular remodeling after stroke. Nat. Commun..

[112] Manuel G.E., Johnson T., Liu D. (2017). Therapeutic angiogenesis of exosomes for ischemic stroke. Int. J. Physiol. Pathophysiol. Pharmacol..

[113] Wang S., Aurora A.B., Johnson B.A., Qi X., McAnally J., Hill J.A., Richardson J.A., Bassel-Duby R., Olson E.N. (2008). The endothelial-specific microRNA miR-126 governs vascular integrity and angiogenesis. Dev. Cell.

[114] Sharghi-Namini S., Tan E., Ong L.L., Ge R., Asada H.H. (2014). Dll4-containing exosomes induce capillary sprout retraction in a 3D microenvironment. Sci. Rep..

[115] Åkerblom M., Sachdeva R., Barde I., Verp S., Gentner B., Trono D., Jakobsson J. (2012). MicroRNA-124 is a subventricular zone neuronal fate determinant. J. Neurosci..

[116] Sun Y., Gui H., Li Q., Luo Z.M., Zheng M.J., Duan J.L., Liu X. (2013). MicroRNA-124 protects neurons against apoptosis in cerebral ischemic stroke. CNS Neurosci. Ther..

[117] Uzdensky A.B. (2019). Apoptosis regulation in the penumbra after ischemic stroke: Expression of pro- and antiapoptotic proteins. Apoptosis.

[118] Deng M., Xiao H., Peng H., Yuan H., Xu Y., Zhang G., Tang J., Hu Z. (2018). Preservation of neuronal functions by exosomes derived from different human neural cell types under ischemic conditions. Eur. J. Neurosci..

[119] Pei X., Li Y., Zhu L., Zhou Z. (2020). Astrocyte-derived exosomes transfer miR-190b to inhibit oxygen and glucose deprivation-induced autophagy and neuronal apoptosis. Cell Cycle.

[120] Chen W., Wang H., Zhu Z., Feng J., Chen L. (2020). Exosome-Shuttled circSHOC2 from IPASs Regulates Neuronal Autophagy and Ameliorates Ischemic Brain Injury via the miR-7670-3p/SIRT1 Axis. Mol. Ther. Nucleic Acids.

[121] Huang W., Liu X., Cao J., Meng F., Li M., Chen B., Zhang J. (2015). miR-134 regulates ischemia/reperfusion injury-induced neuronal cell death by regulating CREB signaling. J. Mol. Neurosci..

[122] Xiao B., Chai Y., Lv S., Ye M., Wu M., Xie L., Fan Y., Zhu X., Gao Z. (2017). Endothelial cell-derived exosomes protect SH-SY5Y nerve cells against ischemia/reperfusion injury. Int. J. Mol. Med..

[123] Huang X., Ding J., Li Y., Liu W., Ji J., Wang H., Wang X. (2018). Exosomes derived from PEDF modified adipose-derived mesenchymal stem cells ameliorate cerebral ischemia-reperfusion injury by regulation of autophagy and apoptosis. Exp. Cell Res..

[124] Liu Y., Fu N., Su J., Wang X., Li X. (2019). Rapid Enkephalin Delivery Using Exosomes to Promote Neurons Recovery in Ischemic Stroke by Inhibiting Neuronal p53/Caspase-3. Biomed. Res. Int..

[125] Liu X., Zhang M., Liu H., Zhu R., He H., Zhou Y., Zhang Y., Li C., Liang D., Zeng Q. (2021). Bone marrow mesenchymal stem cell-derived exosomes attenuate cerebral ischemia-reperfusion injury-induced neuroinflammation and pyroptosis by modulating microglia M1/M2 phenotypes. Exp. Neurol..

[126] Ha D., Yang N., Nadithe V. (2016). Exosomes as therapeutic drug carriers and delivery vehicles across biological membranes: Current perspectives and future challenges. Acta Pharm. Sin. B.

[127] Lakhal S., Wood M.J. (2011). Exosome nanotechnology: An emerging paradigm shift in drug delivery: Exploitation of exosome nanovesicles for systemic in vivo delivery of RNAi heralds new horizons for drug delivery across biological barriers. Bioessays.

[128] Tian T., Zhang H.X., He C.P., Fan S., Zhu Y.L., Qi C., Huang N.P., Xiao Z.D., Lu Z.H., Tannous B.A. (2018). Surface functionalized exosomes as targeted drug delivery vehicles for cerebral ischemia therapy. Biomaterials.

[129] Alvarez-Erviti L., Seow Y., Yin H., Betts C., Lakhal S., Wood M.J. (2011). Delivery of siRNA to the mouse brain by systemic injection of targeted exosomes. Nat. Biotechnol..

[130] Tian T., Cao L., He C., Ye Q., Liang R., You W., Zhang H., Wu J., Ye J., Tannous B.A. (2021). Targeted delivery of neural progenitor cell-derived extracellular vesicles for anti-inflammation after cerebral ischemia. Theranostics.

